# Microarray analysis of genes with differential expression of m6A methylation in lung cancer

**DOI:** 10.1042/BSR20210523

**Published:** 2021-09-17

**Authors:** Shuo Wu, Xing Lv, Yan Zhang, Xi Xu, Feng Zhao, Yao Zhang, Lizhan Chen, Haifeng ou-Yang, Xinyu Ti

**Affiliations:** 1 Department of Pulmonary and Critical Care Medicine, Xijing Hospital, Fourth Military Medical University, Xi’an, Shanxi 710032, China; 2 Department of Pulmonary Medicine, Thoracis Hospital Xi’an International Medical Center, Xi’an, Shanxi 710100, China

**Keywords:** gene ontology, Kyoto Encyclopedia of Genes and Genomes, lung cancer, m6A, microarray analysis

## Abstract

**Purpose:** N6-methyladenosine (m6A) is among the most abundant mRNA modifications in eukaryote. The aim of the present study was to investigate function of m6A mRNA methylation in lung cancer and the underlying mechanism.

**Methods:** Microarray analysis was performed to detect the differences in RNA expression between cancerous and adjacent non-cancerous tissue samples. The target mRNAs were subjected to Gene Ontology (GO) and Kyoto Encyclopedia of Genes and Genomes (KEGG) pathway enrichment analyses. Hierarchical clustering of RNAs was conducted to identify distinct m6A methylation or expression patterns between the samples.

**Results:** In the present study, some differentially expressed genes (DEGs) of mRNAs were identified, including up-regulated secret phosphoprotein 1 (SPP1) and down-regulated pRB. Functional enrichment analysis revealed that while differential hypermethylation was related to cell cycle, intracellular part and protein binding, the main pathway involved herpes simplex virus 1 infection related to down-regulated AKT, Araf1 and BCL2A1. In the meantime, sexual reproduction, cohesin complex and protein C-terminus binding was functionally linked to differential hypomethylation, while fluid shear stress and atherosclerosis were identified as the main pathways related to up-regulated GST and CNP.

**Conclusions:** We showed that lung cancer development involved differential expression of SPP1 and pRB mRNA, as well as m6A mRNA methylation in AKT, APAF1, BCL2A1, GST and CNP genes.

## Introduction

Lung cancer is one of the most common cancers in the world, which is commonly divided into small cell lung cancer (SCLC), lung adenocarcinoma and lung squamous cell carcinoma in non-small cell lung cancer (NSCLC) [1–3]. It is estimated that lung cancer-related deaths will account for 23% of all cancer-related deaths by 2020 [4]. RNA methylation modification accounts for more than 80% of all RNA modifications [5]. In addition to mRNAs, many non-coding RNAs, such as circular RNA and long non-coding RNA (lncRNA), harbor N6-methyladenosine (m6A) methylation. As abundant multifunctional ncRNAs, lncRNAs represent a class of transcripts with a length of more than 200 nt, which display no or limited protein-coding capacity. Classification of lncRNAs largely depends on their functional roles and conservation. And m6A is the most common post-transcriptional modification on eukaryotic mRNAs and lncRNAs [6]. m6A methylation plays an important role in cell differentiation, tissue development and DNA repair [7–9]. In recent years, m6A methylation modification has been used for predicting the prognosis of cancer and regulating cancer cell proliferation, apoptosis and migration [10,11].

It has been shown that decreased m6A mRNA methylation led to down-regulated expression of hepatocyte growth factor (c-met), promoting the drug sensitivity of NSCLC cells in a c-MET-/HGF-dependent manner [12]. Jin et al. [13] found that RNA methyltransferase METTL3-mediated m6A modification can promote mRNA translation to increase the resistance of lung cancer to tumor treatment and metastasis. Lung epichek, a 6-marker panel methylation-based plasma test, has shown strong performance in lung cancer prediction and detection of high proportions of early-stage NSCLC and SCLC, while significantly improving predictive accuracy when combined with the established risk factors [14].

The present study aims to establish the expression profile of lung cancer through m6A microarray detection, to identify the hub mRNAs involved in lung cancer by bioinformatics analysis, and to conduct a control study using human normal lung epithelial cells. Gene Ontology (GO) and Kyoto Encyclopedia of Genes and Genomes (KEGG) pathway enrichment analysis revealed the potential biological functions of target genes.

## Methods

### Samples

Five pairs of cancerous and adjacent non-cancerous tissue samples were obtained from lung cancer patients who were diagnosed by biopsy (Supplementary Table S1). The samples were collected within 10 min after tumor excision, immediately immersed in RNAlater® stabilization solution (Thermo Fisher Scientific, Carlsbad, CA, U.S.A.), and then stored at −80°C until used.

### Microarray and computational analyses

The preparation of cancerous and adjacent non-cancerous samples as well as microarray hybridization were carried out according to Arraystar’s standard protocol. Briefly, the total RNAs were immunoprecipitated with anti-m6A antibodies. The modified RNAs termed as ‘IP’ were eluted from immunoprecipitated magnetic beads, while the unmodified RNAs named as ‘Sup’ were recovered from the supernatants. The ‘IP’ and ‘Sup’ RNAs were labeled respectively with Cy5 and Cy3 as cRNAs in separate reactions using Arraystar Super RNA Labeling Kit (Arraystar Inc., U.S.A.). The cRNAs were combined together and hybridized on to Arraystar Human mRNA and lncRNA Epitranscriptomic Microarray (8×60K). After being washed, the arrays were scanned in two-color channels by an Agilent Scanner G2505C (Agilent, U.S.A.). Agilent Feature Extraction software (version 11.0.1.1) was used to analyze acquired array images. Raw intensities of IP and Sup were normalized with an average of log2-scaled Spike-in RNA intensities.

The m6A methylation level was calculated for the percentage of modification based on normalized intensities of the IP and Sup. The RNA expression level was determined based on the total of normalized intensities of RNA in IP and Sup. Hierarchical Clustering was performed to identify distinct m6A-methylation or expression pattern among samples.

### m6A immunoprecipitation

A total of 1–3 μg of total RNA and m6A spike-in control mixture were added to 300 μl of 1× IP buffer (50 mM Tris/HCl, pH 7.4, 150 mM NaCl, 0.1% NP40, 40 U/μl RNase inhibitor, Thermo Fisher Scientific, U.S.A.) containing 2 μg of anti-m6A rabbit polyclonal antibody (Abcam, U.S.A.). The reaction was incubated with head-over-tail rotation at 4°C for 2 h. Twenty microliters of Dynabeads™ M-280 Sheep Anti-Rabbit IgG suspension per sample (Thermo Fisher Scientific, U.S.A.) was blocked with freshly prepared 0.5% BSA at 4°C for 2 h, washed three times with 300 μl of 1× IP buffer, and resuspended in the total RNA–antibody mixture prepared above. The RNA binding to m6A–antibody-conjugated beads was carried out with head-over-tail rotation at 4°C for 2 h. Then, the beads were sequentially washed three times with 500 μl of 1× IP buffer and twice with 500 μl of Wash buffer (50 mM Tris/HCl, pH 7.4, 50 mM NaCl, 0.1% NP40, 40 U/μl RNase Inhibitor). The enriched RNA was eluted with 200 μl of Elution buffer (10 mM Tris/HCl, pH 7.4, 1 mM EDTA, 0.05% SDS, 40 U Proteinase K, Thermo Fisher Scientific, U.S.A.) at 50°C for 1 h. The RNA was extracted by acid phenol–chloroform (Beyotime, Shanghai) and ethanol precipitated.

### GO analysis and KEGG pathway enrichment analysis

GO annotation includes biological process, cellular component and molecular function analyses. GO analysis was performed using top GO package in R environment for statistical computing and graphics. KEGG enrichment analysis was performed using Ingenuity Pathway Analysis. Pathway analysis associates the differentially m6A-methylated or expressed mRNAs enriched in certain biological pathways. The statistical significance of the enrichment was calculated by Fisher’s Exact test *P*-value and also −log10(*P*) transformed as the enrichment score. The adjusted *P*-value <0.05 and |FC| ≥ 1.5 were recognized as the threshold. Studies have shown that false discovery rate-corrected *P*-values are commonly used as thresholds during bioinformatics analysis. FC indicates the fold change in expression level of the same gene in two samples and is the most basic method to detect differentially expressed genes (DEGs) [15]. The lower the *P*-value or the higher the enrichment, the better the statistical significance of differentially m6A-methylated or expressed mRNAs in that pathway.

### Statistical method

Statistical significance of the enrichments was determined by Fisher’s Exact test *P*-value. A paired *t* test with *P*<0.05 and |FC| ≥ 1.5 were considered statistically significant.

## Results

### Identification of differentially expressed or methylated genes

**Figure 1:**
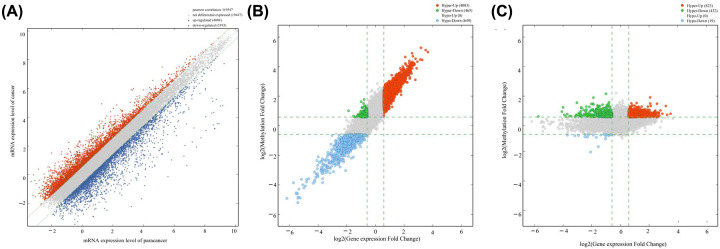
Differential expression or methylation of mRNAs between cancerous and adjacent non-cancerous tissue samples (**A**) Scatter plot of differential expression of mRNAs. (**B**) Scatter plot of differential RNA methylation. (**C**) Scatter plot of differential mRNA methylation.

The expression data of 26644 mRNAs (4004 with up-regulated expression and 3193 with down-regulated expression) were obtained by microarray analysis (Figure 1A). A total of 5117 DEGs were identified in tumor samples and paracancerous tissue samples, of 4003 with hypermethylation were up-regulated, while 465 with hypermethylation were down-regulated (Figure 1B). Moreover, we found that while 823 hypermethylated mRNAs were up-regulated, 432 hypermethylated mRNAs and 19 hypomethylated mRNAs were down-regulated (Figure 1C).

### Hierarchical clustering of differentially methylated mRNAs, lncRNAs and ncRNAs

**Figure 2:**
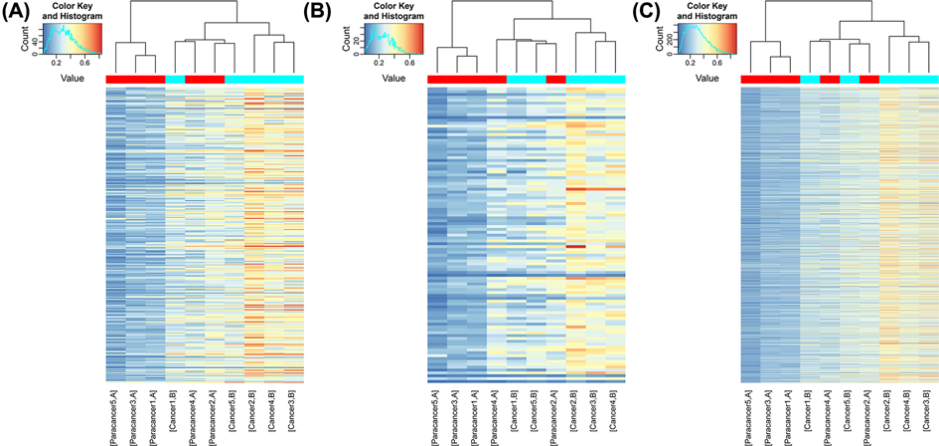
Heat maps of mRNAs, lncRNAs and small ncRNAs that are differentially methylated or expressed (**A**) Differentially methylated lncRNAs. (**B**) Differentially methylated small ncRNAs. (**C**) Differentially methylated mRNAs.

Hierarchical clustering was performed on the mRNA, lncRNA and small ncRNA based on all remarkable methylated to hypothesize the relationship between samples. As depicted in Figure 2, compared with differentially methylated lncRNAs and small ncRNAs, differentially methylated m6A of mRNAs was more widely distributed between cancerous and adjacent non-cancerous samples of lung cancer patients.

Among those differentially expressed mRNAs, ten most significantly up- or down-regulated mRNAs are listed respectively in Tables 1 and 2. Moreover, Table 3 summarizes the ten most significantly differentially m6A-methylated mRNAs.

**Table 1:** Significantly up-regulated mRNAs

Probe name	Gene	FC	RNA length
ASHG19AP1B10007636111	*IBSP*	11.34104903	1546
ASHG19AP1B10002183511	*FN1*	9.023328758	2388
ASHG19AP1B10014106611	*HEPHL1*	7.335276598	5345
ASHG19AP1B12461375211	*SPP1*	6.572333576	1558
ASHG19AP1B12656285411	*MCM10*	6.320731233	4547
ASHG19AP1B13050495511	*SPP1*	6.289492835	1581
ASHG19AP1B11781843811	*COL10A1*	6.105570186	3289
ASHG19AP1B10295406811	*SPP1*	6.088150895	825
ASHG19AP1B10009855511	*PXDN*	6.038281643	6808
ASHG19AP1B10013077611	*KLHL30*	5.864080275	3726

**Table 2:** Significantly down-regulated mRNAs

Probe name	Gene	FC	RNA length
ASHG19AP1B11096563311	*STATH*	0.013766057	534
ASHG19AP1B10022676211	*FDCSP*	0.018860649	566
ASHG19AP1B13664140711	*PRR4*	0.021635726	563
ASHG19AP1B10007740711	*SOX10*	0.021861549	2879
ASHG19AP1B11995032711	*PRR4*	0.022819711	326
ASHG19AP1B13482975311	*PRH1*	0.023358160	837
ASHG19AP1B10826094611	*PRB1*	0.023404414	710
ASHG19AP1B10368539711	*PRB4*	0.024149200	586
ASHG19AP1B11206865311	*PRB2*	0.024664568	1429
ASHG19AP1B11281790311	*PRB1*	0.026254622	775

**Table 3:** Differentially methylated mRNAs

Gene symbol	Regulation	FC	*P*	FDR
*APOBEC3G*	Hyper	4.630148095	0.043522445	0.444782472
*ZIC2*	Hyper	3.776499153	0.041880686	0.444782472
*CXCL10*	Hyper	3.732806269	0.029983185	0.444782472
*PDE4D*	Hyper	3.011888487	0.002013734	0.444782472
*TPD52L1*	Hyper	2.998298855	0.015022040	0.444782472
*NPPC*	Hypo	0.291092116	0.035823834	0.444782472
*ZBTB16*	Hypo	0.374241455	0.016901284	0.444782472
*SCML4*	Hypo	0.577834899	0.019203801	0.444782472
*SULT1E1*	Hypo	0.587708474	0.038919508	0.444782472
*ASXL3*	Hypo	0.593221489	0.010401243	0.444782472

### Functional enrichment analysis of DEGs

**Figure 3:**
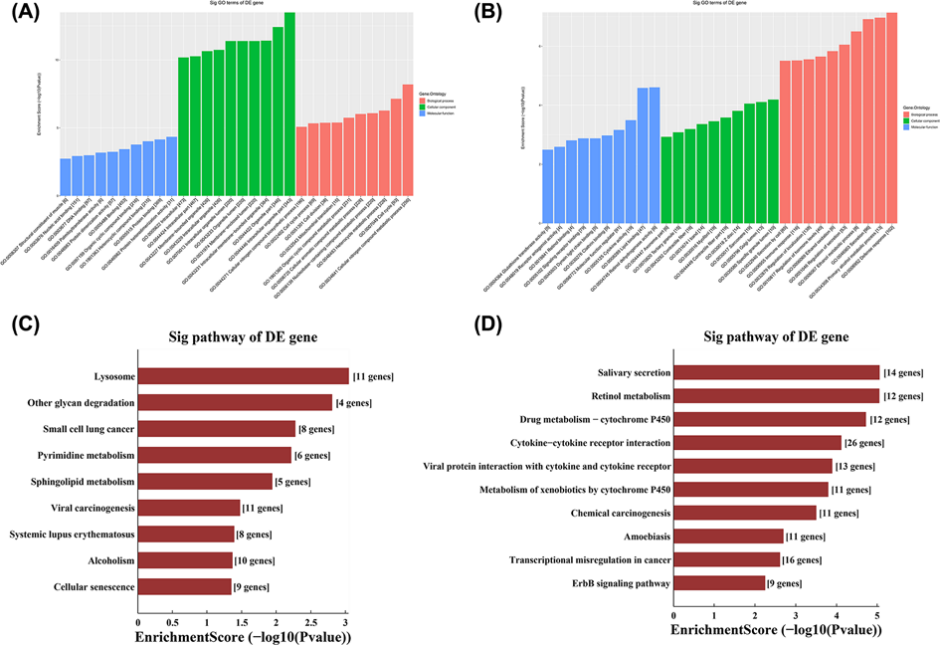
GO and KEGG analyses of differentially expressed mRNAs (**A**) The top ten enriched items obtained from GO analysis of up-regulated mRNAs with differential expression. (**B**) The top ten enriched items obtained from GO analysis of down-regulated mRNAs with differential expression. (**C**) The first ten enriched pathways identified in KEGG analysis of up-regulated mRNAs with differential expression. (**D**) The first ten enriched pathways identified in KEGG analysis of down-regulated mRNAs with differential expression.

As shown in Figure 3A, significantly up-regulated mRNAs with differential expression were mainly distributed in intracellular organelle part and participate in the metabolism of cellular nitrogen compound metabolic process, mainly protein heterodimerization activity and protein binding. Likewise, the significantly down-regulated mRNAs were mainly distributed in specific granule lumen, Golgi lumen and sarcomere, and participates in the defense response, mainly retinol dehydrogenase activity and lipid binding (Figure 3B). The KEGG results were shown in Figure 3C, while the main enriched pathway for up-regulated mRNAs involved lysosome, retinol metabolism-related pathway was mainly enriched in down-regulated mRNAs (Figure 3D).

### Functional enrichment analysis of differentially methylated mRNAs

**Figure 4:**
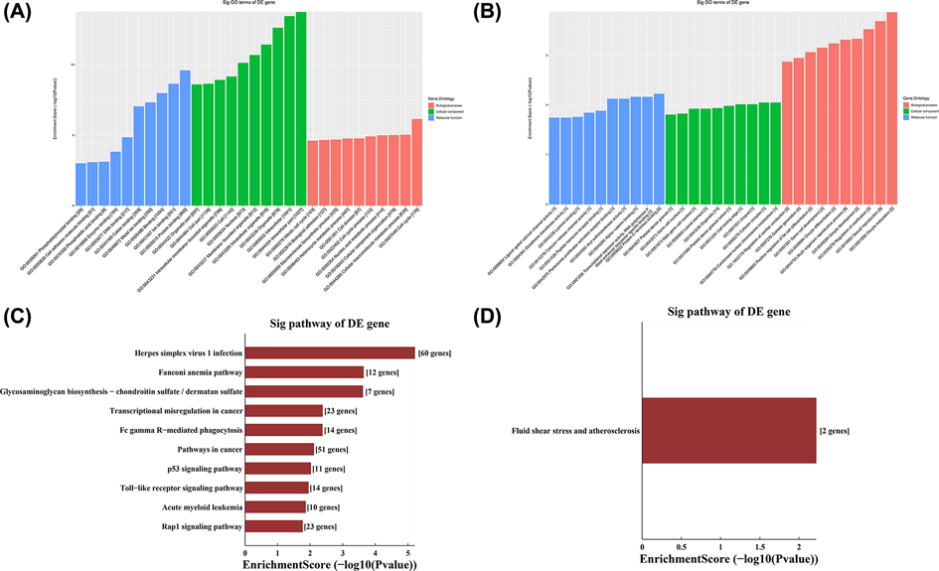
GO and KEGG analyses of differentially methylated mRNAs (**A**) The top ten enriched items obtained from GO analysis of differentially hypermethylated mRNAs. (**B**) Top ten enriched items obtained from GO analysis of differentially hypomethylated mRNAs. (**C**) The first ten enriched pathways identified in KEGG analysis of differentially hypermethylated mRNAs. (**D**) The top ten enriched pathways identified in KEGG analysis of differentially hypomethylated mRNAs.

As shown in Figure 4A, differentially hypermethylated mRNAs were mainly distributed in the intracellular part and participated in the cell cycle, mainly protein binding and ion binding. Meanwhile, differentially hypomethylated mRNAs were mainly distributed in the adhesin complex and concentrated nuclear chromosome mitochondria, and participate in the oocyte maturation and sexual reproduction, mainly protein C-terminal binding (Figure 4B).

### Functional pathway analysis of differentially methylated mRNAs

KEGG analysis revealed that herpes simplex virus 1 infection-related pathway was mainly enriched in the ten most significantly hypermethylated mRNAs (Figure 4C), while the main enriched pathways among the ten most significantly hypomethylated mRNAs involved fluid shear stress and atherosclerosis (Figure 4D).

## Discussion

An increasing number of studies have shown that the diagnostic accuracy of lung cancer screening can be improved by detecting DNA methylation of lung cancer-specific genomes in sputum and plasma [16–19]. However, the molecular mechanism underlying the progression of lung cancer remains unclear. Therefore, it is very important to investigate the mechanism while identifying the molecular targets for diagnosis and treatment. LncRNAs are dysregulated in all aspects of cell homeostasis and involved in a variety of cellular and biological processes including proliferation, apoptosis, migration, invasion, metastasis, chromatin remodeling, gene transcription and post-transcriptional processing [20]. The basic role of small ncRNAs in many human diseases has also been determined. It has been shown that small ncRNAs play an important role in cancer development and progression as well as drug resistance [21]. In this study, genes associated with mRNAs, lncRNAs and small ncRNAs were identified respectively at the adjusted *P*<0.05 and |FG| ≥ 1.5. Given that differential expression and methylation analysis did not reveal the genes corresponding to lncRNAs and small ncRNAs, GO and KEGG pathway analyses were conducted to analyze gene function of the selected differentially methylated mRNAs.

Notably, among the significantly up-regulated mRNAs, secret phosphoprotein 1 (SPP1), also known as osteopontin, is a secreted acidic glycoprotein with multiple functions, which is overexpressed in many types of cancer [22,23]. It has been reported that this glycoprotein is related to invasive phenotype and TNM stage of lung cancer [24]. SPP1 expression was previously found to be up-regulated in lung cancer tissues [24]. The similar results were observed in the present study. Here, the KEGG analysis identified lysosome as the most abundant differential expression pathway. Li et al. suggested that lysosome-associated membrane protein 3 (lamp3) may be involved in tumor invasion and metastasis by regulating the downstream signaling pathway of SPP1 [25]. Moreover, it has been demonstrated that lysosome-related drugs may be linked to chemosensitizers and immunomodulators in cancer chemotherapy, and can enhance immune response by reversing the chelation of drugs in lysosome, thus playing a role in inhibiting lung cancer cells [26]. Notably, demethylation treatment can enhance the expression of lysosomes in lung cancer cells, possibly facilitating the development of lysosomal targeting or demethylation drugs for lung cancer treatment [14]. Besides, autophagy inhibitors can serve as potential antitumor drugs through inhibiting autophagy lysosome fusion [27]. In the present study, we provided more experimental data supporting the above observations.

Inactivation of tumor suppressor pRB may contribute to the occurrence and development of tumors [28]. The KEGG analysis showed that pRB was mainly concentrated in the down-regulated SCLC pathway. Similarly, we observed a significant down-regulation of pRB in A549 cell line. Deletion of RB1 can effectively transform neuroendocrine (NE) and alveolar type 2 (SPC) cells, leading to SCLC [29]. Moreover, it has been shown that RB mutation may serve as an indicator for SCLC transformation of EGFR mutant NSCLC [30]. This study presented valuable findings with regard to pRB.

In the present study, GO annotations of the differentially methylated genes revealed that gene hypermethylation was related to cell cycle, intracellular part and protein binding, while sexual reproduction, cohesin complex and portein C-terminus binding were associated with gene hypomethylation. Moreover, the KEGG enrichment analysis identified herpes simplex virus 1 infection as the main pathway of differential hypermethylation. Strikingly, while a number of genes including Akt, Apaf1, BCL2A1, TSC1, PML, IRAK4, BCL2, CARD9 and IFN-α were down-regulated based on the down-regulation sites shown in the pathway map (Supplementary Figure S1), the methylation difference decreased accordingly. Akt, generally referred to as protein kinase B, plays a key role in cell proliferation, survival and metabolism, and the overactivation of Akt is associated with the cancer progression [31]. Previous studies have shown that decreased m6A methylation in cancer cells can activate the Akt pathway, resulting in increased cell proliferation and carcinogenicity [32]. This observation was similar with our data in this study. Regulation of Akt activation and autophagy induction were found to be significantly related to the treatment and prognosis of lung cancers such as NSCLC, lung squamous cell carcinoma and lung adenocarcinoma [33–35]. Apaf1 and autophagy induction could underlie the recovery process of cell [36]. Apaf1 methylation is significantly associated with its mRNA expression inhibition [37], and detection of apaf1 methylation status in the blood sample can be applied for cancer diagnosis [38].

BCL2A1 is a member of Bcl-2 protein family that forms heterodimer or homodimer, acting in tumorigenesis as an anti-apoptotic regulator. The protein encoded by this gene can reduce the release of pro-apoptotic cytochrome *c* into mitochondria, blocking the caspase activation. It has been shown that shear regulators can selectively induce BCL2A1-dependent tumor cells, leading to apoptosis of NSCLC cells [39], while targeting the intrinsic pathway of BCL-2-related apoptosis can effectively treat lung adenocarcinoma [40]. In our study, the expression of BCL2 and BCL2A1 in the differential hypermethylation pathway was down-regulated in A549 cancer cell line compared with normal lung epithelial cells. Zhao et al. reported that the methylation frequency of BCL2 gene in stage I NSCLC was significantly higher than that in non-cancerous lung disease [41]. However, the mechanism of BCL2A1 methylation in NSCLC remains to be studied.

In the present study, we identified fluid shear stress and atherosclerosis as the main enriched pathways of differential hypomethylation related to up-regulation of GST and CNP genes in anti-atherogenesis (Supplementary Figure S2). Endothelial cells convert the friction force (fluid shear stress) from blood flow into biochemical signals that regulate gene expression and cell behavior via certain mechanisms and pathways. Similarly, the above mechanisms underlie atherosclerosis [42]. GST polymorphism may affect enzyme activity, being involved in atherosclerosis [43]. CNP is an autocrine and paracrine mediator released by endothelial cells, cardiomyocytes and fibroblasts, which can regulate important physiological functions of the cardiovascular system, as well as atherosclerosis [44]. CNP can improve lung fibrosis by inhibiting TGF-β signal transduction and myoblast differentiation [45]. Given that cardiovascular diseases may affect the treatment outcome of NSCLC [46], we speculate that the expression of GST and CNP could potentially serve as markers for the diagnosis of atherosclerotic complications in NSCLC patients.

In addition, the KEGG analysis showed that the transcriptional misregulation in cancer pathway was an enrichment pathway shared by differential expression down-regulation and differentially hypermethylated mRNA. Transcription is the most important way of RNA biosynthesis. Errors in the process of mRNA transcription could result in production of abnormal proteins, possibly inducing tumor formation [47]. It has been shown that methylation can affect the gene expression, apoptosis and autophagy in lung cancer, thereby altering the tumor staging, metastasis and invasion [48]. The relationships among genes, apoptosis and autophagy in cancer cells need to be further studied.

In conclusion, we identified several mRNAs with differential methylation or differential expression between cancerous and adjacent non-cancerous samples of lung cancer patients by microarray analysis, and further annotated their functions using bioinformatics approaches. We will collect more clinical samples and validate these findings in the future studies.

## Supplementary Material

Supplementary Figures S1-S2 and Table S1

## Data Availability

The authors agree on sharing the present study’s data and its deposit in public repositories, upon reasonable request.

## Abbreviations

cRNA: complementary RNA
DEG: differentially expressed gene
GO: Gene Ontology
HGF: hepatocyte growth factor
IP: Internet Protocol
KEGG: Kyoto Encyclopedia of Genes and Genomes
lncRNA: long non-coding RNA
m6A: N6-methyladenosine
ncRNA: non-coding RNA
NSCLC: non-small cell lung cancer
SCLC: small cell lung cancer
SPP1: secret phosphoprotein 1
TNM: lymph node metastasis
