# Supplementary material for: Microarray analysis of genes with differential expression of m6A methylation in lung cancer
Source: Biosci Rep. 2021 Sep 17;41(9):BSR20210523. doi: 10.1042/BSR20210523 (PMC8450313; doi:10.1042/BSR20210523)
Supplement: Supplementary Figures S1-S2 and Table S1 [file BSR-2021-0523_supp.pdf]

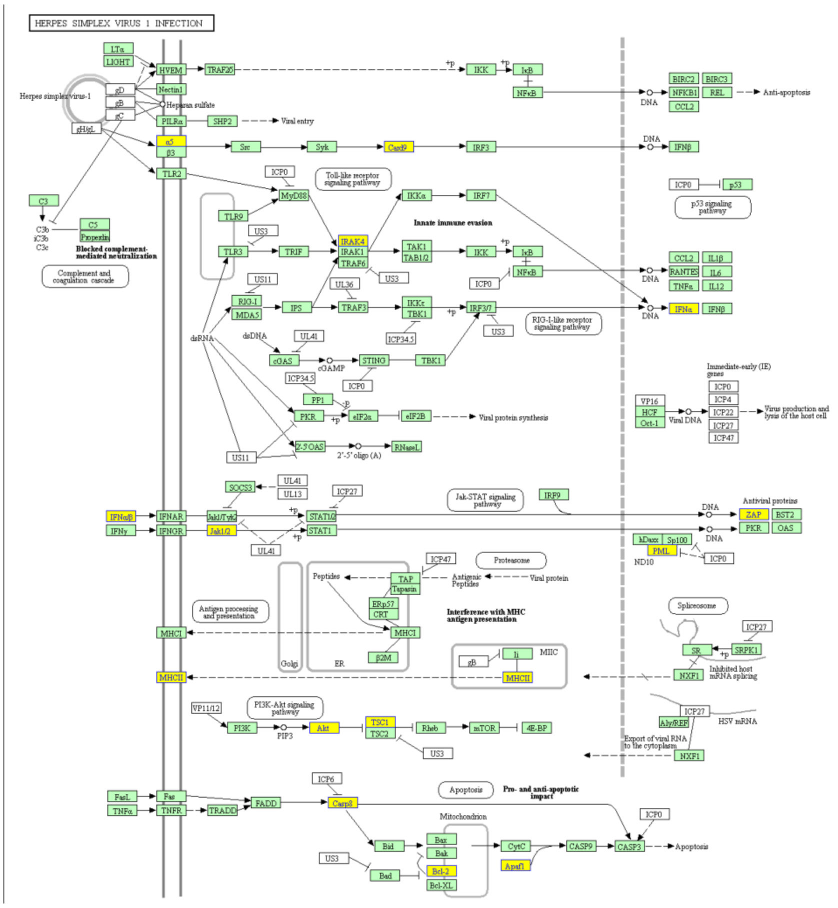

Supplementary Figure. 1 Path diagram of herpes simplex virus 1 infection pathway with differential hypermethylation

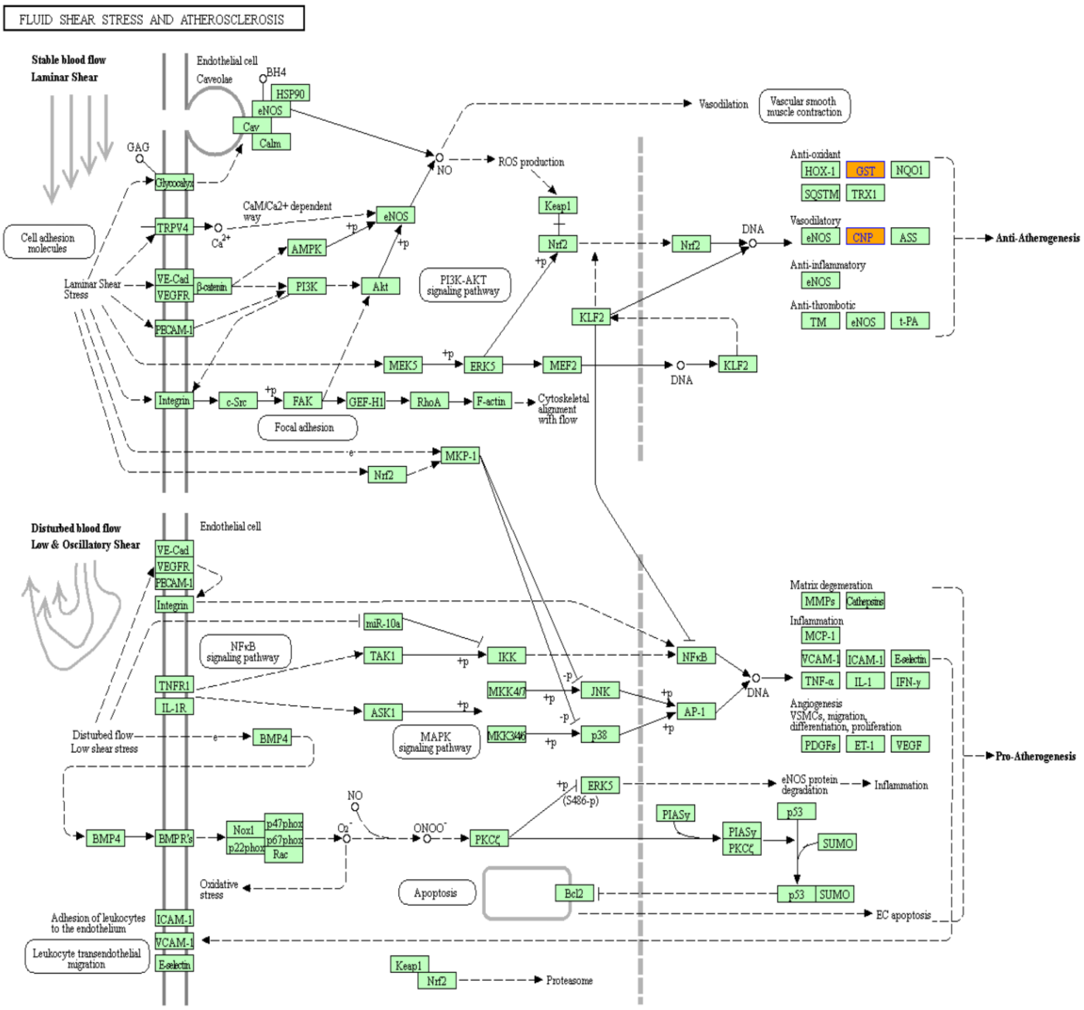

Supplementary Figure. 2 Path diagram of fluid shear stress and atherosclerosis pathway with differential hypomethylation

Supplementary Table 1 Biopsy results of patient

Number	Gender	Age	ID	Type
1	Female	59	80416184	Lung adenocarcinoma
2	Male	74	1800986509	Non-small cell lung cancer
3	Male	58	1800983146	Squamous lung cancer
4	Male	47	1800982648	Non-small cell lung cancer
5	Female	41	1800992593	Small cell lung cancer
